# Molecular insights on ar-turmerone as a structural, functional and pharmacophoric analogue of synthetic mosquito repellent DEET by comprehensive computational assessment

**DOI:** 10.1038/s41598-022-19901-2

**Published:** 2022-09-16

**Authors:** Priyashi Rao, Dweipayan Goswami, Rakesh M. Rawal

**Affiliations:** 1 Department of Biochemistry & Forensic Science, University School of Sciences, Gujarat University, Ahmedabad, Gujarat 380009 India; 2 Department of Microbiology & Biotechnology, University School of Sciences, Gujarat University, Ahmedabad, Gujarat 380009 India; 3 Department of Life Science, University School of Sciences, Gujarat University, Ahmedabad, Gujarat 380009 India

**Keywords:** Molecular modelling, Computational biology and bioinformatics

## Abstract

Mosquitoes are vectors for a variety of infectious illnesses, and chemical synthetic insecticides have made it possible to control them effectively. Mosquito repellents are a typical means of keeping mosquitos at bay. Because of its main effectiveness of skin permeability, *N*,*N*-Diethyl-meta-toluamide (DEET) is one of the most extensively used mosquito repellents but a dangerous synthetic chemical. DEET was identified about a decade ago to inhibit mosquito's Odorant Binding Protein 1 (OBP1), impairing the mosquito's ability to recognise the host body odour. OBP1 has been identified as a possible target for the development of new mosquito repellents since its discovery. Essential oils from different plants, on the other hand, have been used to repel mosquitos since antiquity. One essential oil from the *Curcuma longa* (Zingiberales: Zingiberaceae) rhizome display mosquito repellent properties, according to the literature. Furthermore, one of the phytochemicals found in abundance in *C. longa* essential oil, ar-turmerone, exhibits mosquito repellency as comparable to synthetic DEET. Till date studies on *in-silico* interaction of natural ar-turmerone with OBP1, which we depict in our current work are scarce. Further, there exist no published reports demonstrating the literary evidence on detailed insights of interaction of DEET with OBP1 along with Molecular Dynamics (MD) simulation studies. We further performed detailed molecular investigations using pharmacophore analysis of ar-turmerone and compared it with DEET, where our findings in the current manuscript unveils for the first time that ar-turmerone is a functional, structural and pharmacophoric analogue of DEET.

## Introduction

*N*,*N*-Diethyl-*meta*-toluamide, broadly referred as DEET or diethyltoluamide is one of the most widely used chemical compound in commercially available mosquito repellents. DEET was developed in the year 1944 for the United States Army by Samuel Gertler during the World War II to protect soldiers at jungle warfare^1^. For more than 40 years now, DEET is being used in abundance to repel mosquitoes, ticks, and many other biting insects by applying on skin or cloths in various mixtures as spray or lotion. The concentration of DEET in commercial mosquito repellent formulation is found to be as low as 10% and as high as 100%. Use and overuse of DEET in various forms have two major concerns, (i) the mosquitoes and insects are gradually acquiring resistance where it has been reported that *Aedes aegypti* (Diptera: Culicidae) have evolved and inherited insensitivity to DEET^2,3^ and (ii) DEET being fat soluble has ability to penetrate skin and enter blood thereby raising toxicity concerns^4^. Consequently, with its profound use at sites near agricultural land and water bodies, their concentrations in aqueous samples from all around the world (e.g., drinking water, streams, open seawater, groundwater and treated effluent) are recorded to range from 40 to 3000 ng/L, highlighting a preliminary environmental concern and a plausible ecological^5^.

Initial evidences of insect OBP-DEET interactions were found in *Anopheles gambiae* (Diptera: Culicidae) Odorant Binding Protein 1 (AgamOBP1) in the year 2012 (by Tsitsanou et al.)^6^ and then in the year 2013 (by Tsitsanou et al.)^7^ respectively suggesting OBP1 to be the protein target of DEET and conceivably for other repellents as well. Once this protein was identified as molecular target for DEET, several reports on interaction of other repellents to this protein in different mosquitoes and insects were published^8,9^. For instance, 1,5‑diphenyl pent‑4‑en‑1‑one derivatives too were reported to interact with OBP1^10^. Today, researchers are emphasizing on developing new bio-inspired repellents against mosquitoes using chemical structure of DEET as the start point by derivatizing DEET and assessing its interaction with OBP1 as one of the ways to overcome the issue of toxicity imposed by synthetic mosquito and insect repellents. Another way by which researchers are trying to tackle the issue of repellent toxicity is to identify natural molecules that can replace these synthetic ones. Till date not many natural analogues of DEET are reported and henceforth, this manuscript shares one such poignant piece of information.

There are multiple evidences that molecules from natural sources show mosquito repellency^11^, as per comprehensively collected data in the report by Benellia and Pavela there are about 110 essential oils that show mosquito repellency^12, 13^. It is reported that volatile phytochemicals from the plant *Ocimum sanctum L.* (Lamiales: Lamiaceae) interact with OBP1 (protein PDB ID: 3N7H and 3Q8I) of *A. gambiae* to show mosquito repellent activity, further phytochemicals 2-Hexadecen-1-ol, DL-alpha-tocopherol, Catechol Phytol, 2-Hydroxy-6-methylbenzaldehyde, monoacetin, lycopersin, and gamma-sitosterol showed efficient binding with OBP1^14^. In a similar study, essential oils from *Vitex negundo L.* (Lamiales: Lamiaceae) have efficacy to interact with OBPs of *A. aegypti*, *Anopheles stephensi* (Diptera: Culicidae), and *Culex quinquefasciatus* (Diptera: Culicidae). Another similar study evaluated some of the volatile natural phytochemicals namely α-pinene, linalool, cis-sabinene hydrate citronellal, verbenone, bornyl acetate α-phellandrene α-terpinene, sabinene, β-pinene, myrcene, p-cymene from *O. sanctum*, and it was observed the myrcene, linalool and α-pinene showed mosquito repellent ability at par with DEET^15^. In another study, plant extract of *Artemisia nilagirica* (Asterales: Asteraceae) is shown to have mosquito larvicidal, pupicidal, adulticidal, and repellent activity against *A. stephensi* and *A. aegypti*, however these previously mentioned studies lack in silico proteomics validation to be able to visualise the probable interaction of essential phytochemicals from the studied plant extract with that against OBP^16^. Similarly, *Nepeta cataria* (Lamiales: Lamiaceae) essential oils and isolated nepetalactones are reported to have mosquito repellent activity against *A. aegypti*, but display no evidences of their interaction with OBP^17^. Zographos et al. in 2017 have represented the significance of assessing the interaction of molecules with OBPs for potential repellent activity which in modern day should be the tool to identify newer mosquito repellents^18^ and later in 2021 this claim was consolidated by Okoli et al.^15^.

Of all the plants studied so far, different phytochemicals from *Curcuma longa* (Zingiberales: Zingiberaceae) are well established to possess mosquito larvicidal, pupicidal, adulticidal, and repellent activity^19^. Curcumin and demethoxycurcumin are well reported to have larvicidal activity against dipterans^20^. Phytochemicals of such as curcumin, demethoxycurcumin, other curcuminoids and ar-turmerone of *C. longa* are well reported to possess Acetylcholine esterase (AChE) and Butyrylcholine esterase (BChE) activity^21–25^, which are key molecular targets for inducing larval mortality^25^. To add to these, in natural mosquito repellent formulations, extract of *C. longa* and its essential oils are found to have inevitable role in mosquito repellent activity^26,27^. Mosquito repellent activity of *C. longa* essential oil was first established in the year 1982 by Helen Su et al., where the analysis of oil showed ar-turmerone to be the primary volatile phytochemical to exhibit mosquito repellence^28^. Later, in the year 2015, Abbas Ali et al. compared the mosquito repellent and mosquito biting deterrent activity of ar-turmerone with DEET and showed ar-turmerone was as effective as DEET in its action at identical concentrations^29^. Thus, there are sufficient claims on curcuminoids including ar-turmerone to possess mosquito repellent activity.

However, despite there being sufficient proofs of ar-turmerone and other curcuminoids to possess mosquito repellent activity, to the best of our knowledge there are no evidence showcasing their molecular interactions with dipteran OBP. Therefore, under current study, we compiled a dataset all the phytochemicals from *C. longa* as per Indian Medicinal Plants, Phytochemistry And Therapeutics (IMPPAT; https://cb.imsc.res.in/imppat/home) library database^30^, which included dihydrocurcumin, bisdemethoxycurcumin, ar-turmerone, tetrahydro bisdemethoxycurcumin, piperine, zingiberene, 2-methylisoborneol, butylated hydroxytoluene, borneol, desmethoxycurcumin, sabinene, alpha-phellandrene, butylhydroxyanisole, cineol and curlone amongst others, segregated them on their nature as volatile and non-volatile, followed by assessing their interaction with OBP1 of *C. quinquefasciatus* and *A. aegypti*. The interaction of DEET with OBP1 was taken as reference. Upon performing molecular docking, it was observed that ar-turmerone in the volatile group of compounds showed best binding with OBP1. Further, analysis of pharmacophore mapping showed that of all the compounds, only ar-turmerone has identical pharmacophore features to those of DEET, making it a structural, functional and pharmacophoric natural analogue of DEET. This finding was further validated by performing Molecular Dynamics (MD) simulations of (i) DEET-OBP1 and (ii) ar-turmerone-OBP1 followed by comparing their interaction profiles. The entire assessments were done on two OBP1 proteins one from *C. quinquefasciatus* and another from *A. aegypti* to consolidate the findings for dipteran OBP’s.

## Results

### Docking assessment of DEET and *C. longa* phytochemicals with OBP1

**Figure 1:**
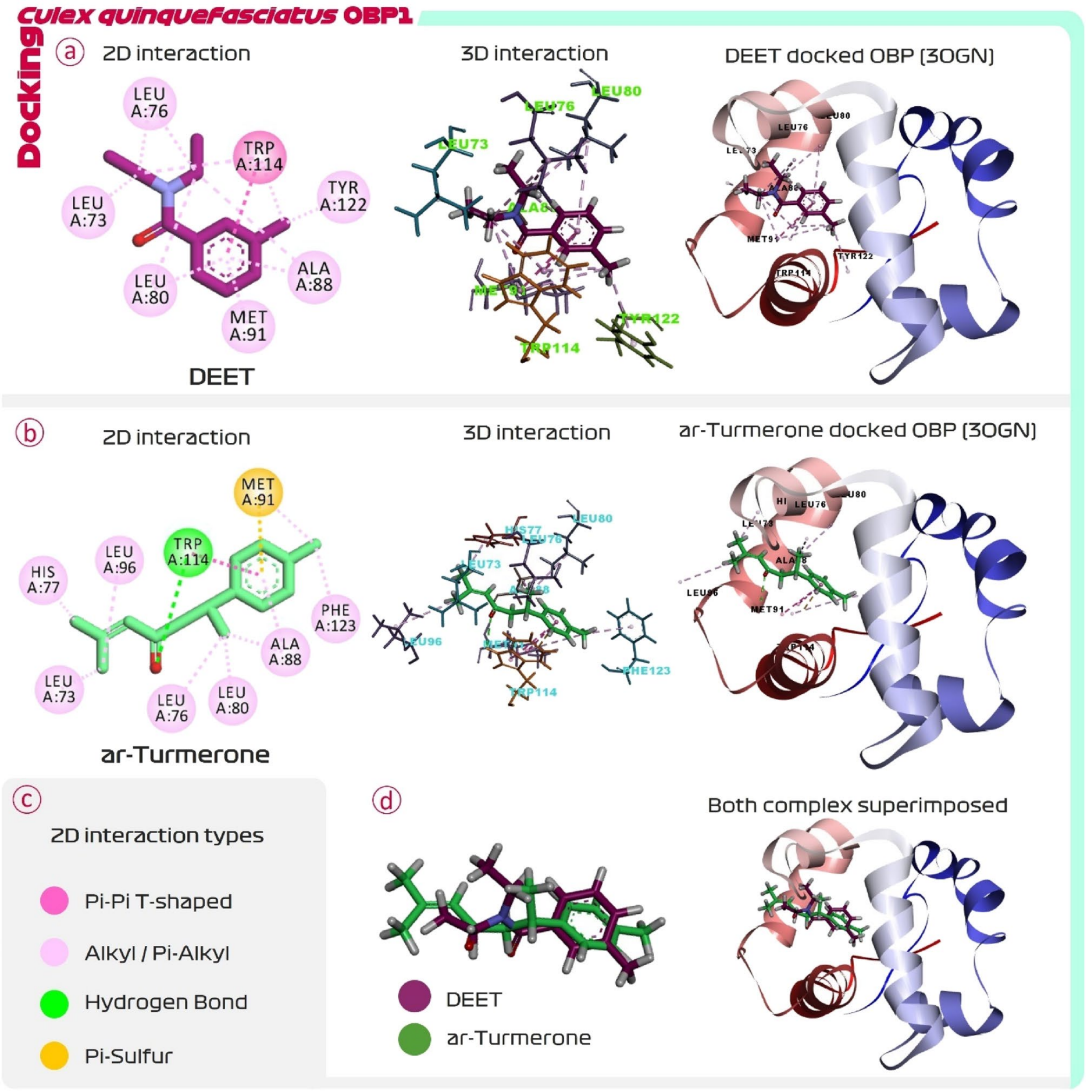
Molecular docking of (**a**) DEET and (**b**) ar-turmerone with OBP1 (3OGN) of *C. quinquefasciatus,* (**c**) interaction types and (**d**) superimposition of docked DEET and ar-turmerone.

DEET is the reference mosquito repellent, and it is well known to interact with OBP1. This interaction of DEET with OBP1 of *C. quinquefasciatus* (3OGN) is considered as reference control. Interaction of DEET with OBP1 show that, DEET recruits TRP114 forming pi–pi T-shaped interaction. From previous studies, this interaction is found to be of huge importance to inhibit the activity of OBP1 which we have discussed in the section of Discussion. This is interaction is formed by aromatic ring of DEET and ketone functional group. The aromatic ring of DEET further recruits Leu80, Ala88 and Met91 all by forming alkyl/pi-alkyl interactions. The methyl group of aromatic ring further interact with Tyr122. The methyl groups attached to nitrogen interacts with Leu73 and Leu76 by forming alkyl/pi-alkyl interactions. All the interactions formed by DEET with OBP1 tends to be hydrophobic in nature and there exists no formation of hydrogen (H) bonds between this ligand and protein (Fig. 1). The binding energy of this interaction is found to be − 6.81 kcal/mol (Table 1).

**Table 1:** List of phytochemicals from *C. longa*, their nature, binding energy, and pharmacophore features.

PubChem CID	Ligand name	Nature of compound	Docking binding energy (kcal/mol) 3OGN	Docking binding energy (kcal/mol) 3K1E	Pharmacophore Features
4284	Diethyltoluamide (DEET)	Volatile	− 6.81	**− 7.11**	**A(1) H(2) H(3) H(4) R(5)**
10,429,233	Dihydrocurcumin	Non-volatile	**− **11.21	**− **11.85	A(4) H(8) H(–) H(–) R(11)
5,315,472	Bisdemethoxycurcumin	Non-volatile	**− **10.68	**− **11.02	A(4) H(–) H(–) H(–) R(8)
160,512	ar-Turmerone	Volatile	**− **9.043	**− 10.23**	**A(1) H(2) H(3) H(4) R(6)**
9,796,792	Tetrahydrobisdemethoxycurcumin	Non-volatile	**− **8.78	**− **9.11	A(1) H(–) H(–) H(–) R(8)
638,024	Piperine	Volatile	**− **7.82	**− **7.54	A(3) H(5) H(4) H(–) R(–)
92,776	Zingiberene	Volatile	**− **7.75	**− **7.44	A(–) H(4) H(3) H(2) R(–)
16,913	2-Methylisoborneol	Volatile	**− **7.59	**− **6.87	A(1) H(–) H(8) H(4) R(–)
31,404	Butylated hydroxytoluene	Volatile	**− **7.41	**− **6.81	A(1) H(3) H(5) H(–) R(6)
64,685	Borneol	Volatile	**− **7.25	**− **6.62	A(1) H(**–**) H(5) H(3) R(–)
5,469,424	Demethoxycurcumin	Non-volatile	**− **7.12	**− **6.56	A(3) H(9) H(–) H(–) R(10)
10,887,971	sabinene	Volatile	**− **6.59	**− **6.55	A(–) H(–) H(2) H(3) R(–)
443,160	alpha-Phellandrene	Volatile	**− **6.52	**− **6.48	A(–) H(–) H(2) H(3) R(–)
24,667	Butylhydroxyanisole	Volatile	**− **5.34	**− **6.21	A(2) H(5) H(–) H(–) R(6)
2758	Cineol	Volatile	**− **4.35	**− **6.01	A(1) H(–) H(4) H(6) R(–)
196,216	Curlone	Volatile	**− **2.45	**− **5.64	A(1) H(4) H(2) H(3) R(–)

A = proton acceptor, H = hydrophobic, and R = aromatic.

Docking of phytochemicals of *C. longa* with OBP1 showed that top three compounds that based on binding energy from docking are dihydrocurcumin, bisdemethoxycurcumin, and ar-turmerone with the binding energies − 11.21, − 10.68, and − 9.043 kcal/mol respectively. Dihydrocurcumin, and Bisdemethoxycurcumin are non-volatile in nature hence its interaction with OBP1 is low, but ar-turmerone is volatile in nature and is reported to have strong mosquito repellent potentials. Therefore, ar-turmerone being volatile and showing strong ability to interact with OBP1 based on XP docking score of − 9.043 kcal/mol is a definite lucrative lead molecule from the lot of curcuminoids. The docking score obtained for all the curcuminoids under study is represented in Table 1. Interaction of ar-turmerone with OBP1 (3OGN) is represented in Fig. 1. Like DEET, ar-turmerone too, have aromatic ring that interacts with Trp114 by making pi-pi t-shaped interaction, the ketone group further interacts with Trp114 by making H-bond. This interaction further strengthens the interaction of Trp114 with the contact ligand. Trp114 is the amino acid of primary importance in interaction of any compound to show specificity with OBP1. Aromatic ring of ar-turmerone interacts with Ala88 by making alkyl/pi-alkyl interaction and with Met91 by forming pi-sulphur interaction. Like DEET, ar-turmerone’s aromatic ring possess methyl group and here it interacts with Phe123. Ar-Turmerone further interacts with Leu73, Ley76, His77, Leu80, and Leu96 by making alkyl/pi-alkyl interaction. ar-turmerone in docking assessment seems to interact with almost all the identical amino acids as by DEET (Fig. 1). Further, the spatial orientation of DEET and ar-turmerone while interacting with OBP1 was determined by superimposing the docked poses of both the ligands. It was observed that both the compounds interact with OBP1 in identical spatial configuration. When the entire experiment was repeated with OBP1 of *Aedes aegypti*, the docking scores for ligands, identical findings were obtained for both dipteran proteins (Table 1).

### Pharmacophore feature mapping of DEET with its analogue ar-turmerone

**Figure 2:**
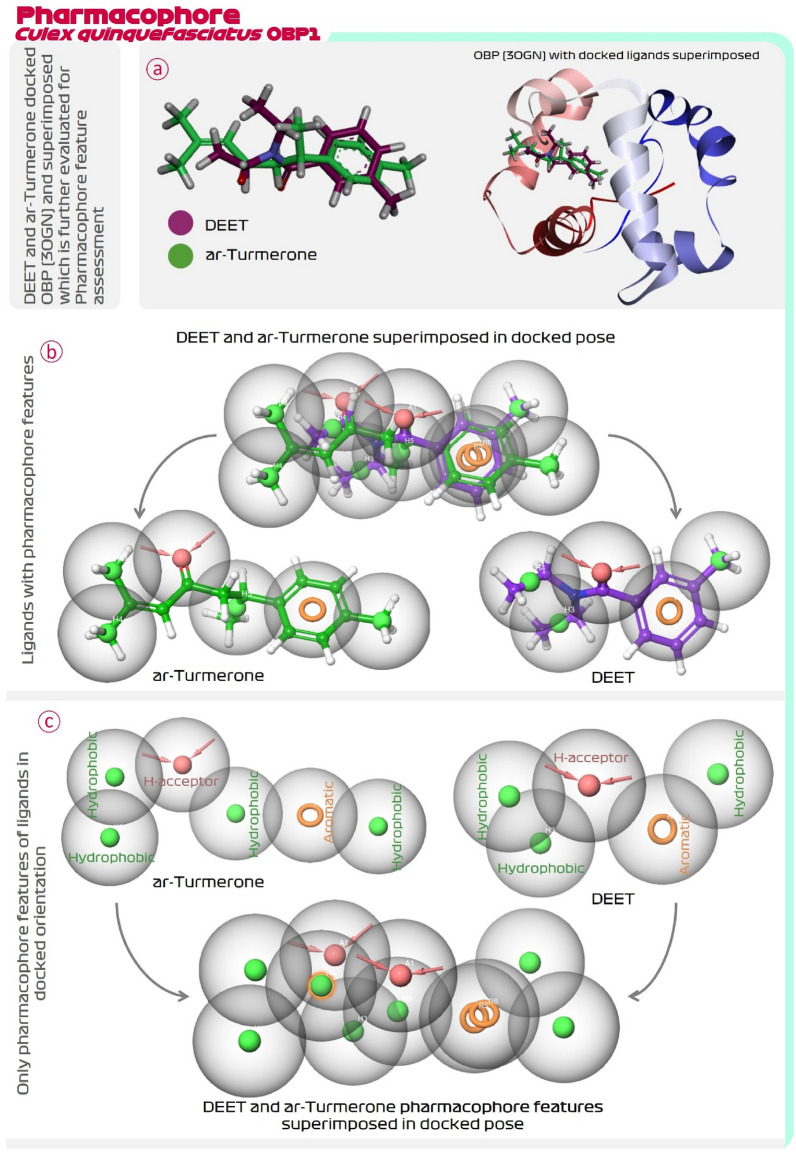
(**a**) Superimposition of docked DEET and ar-turmerone with OBP1 (3OGN) of *C. quinquefasciatus,* (**b**) Pharmacophore feature mapping of DEET and ar-turmerone with ligands displayed and (**c**) Pharmacophore feature mapping of DEET and ar-turmerone without ligands displayed.

**Figure 3:**
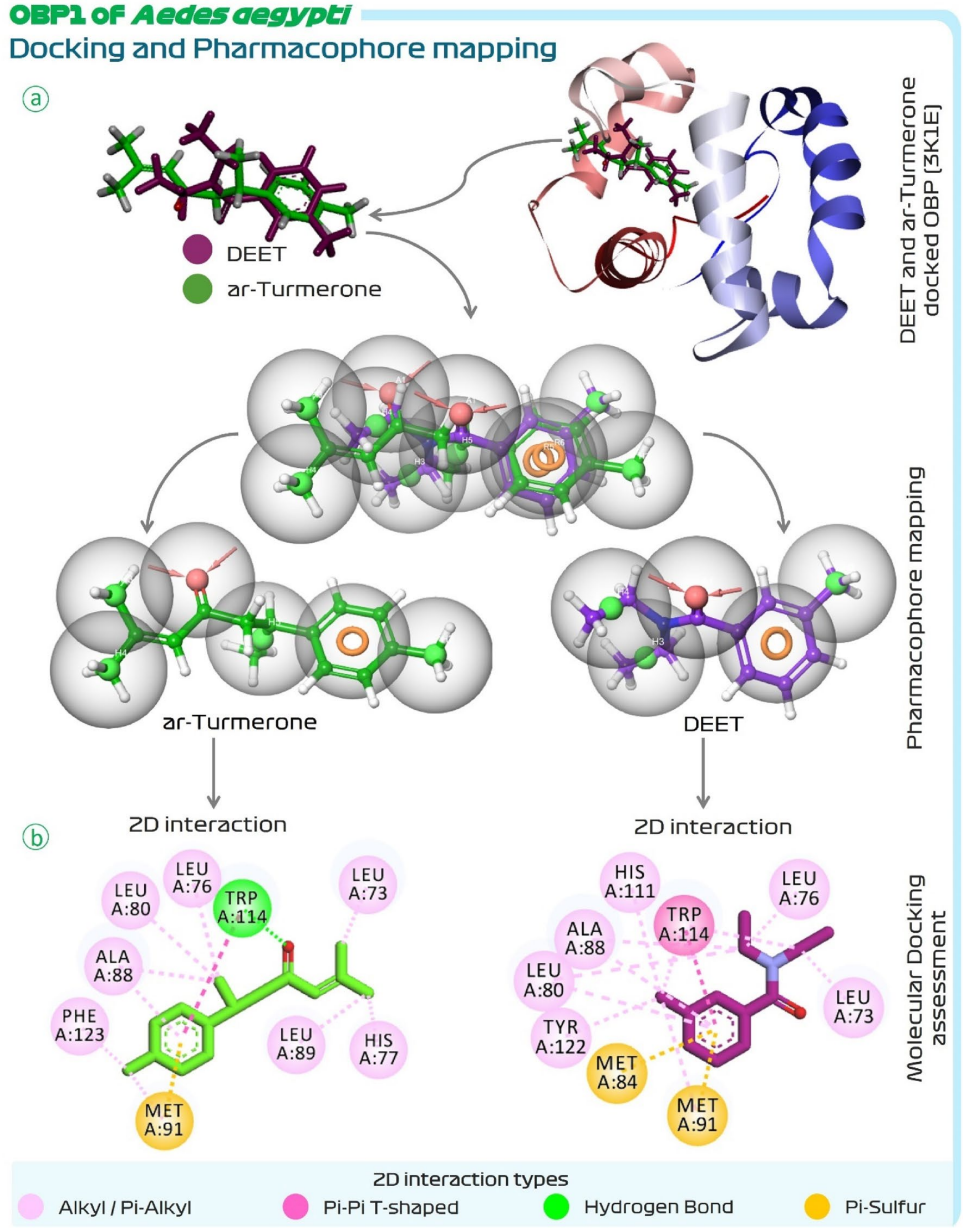
(**a**) Pharmacophore feature mapping and (**b**) molecular docking of DEET and ar-turmerone with OBP1 (3K1E) of *A. aegypti.*

E-pharmacophore feature assessment of DEET suggested that this compound possessed one aromatic pharmacophore feature, one proton acceptor and three hydrophobic features. Thus, DEET in total possess five pharmacophore features. The other compounds from *C. longa* were screened to possess all five of these features and their results are represented in Table 1. It was observed that only ar-turmerone had all the pharmacophore features as found by DEET (Table 1). This makes ar-turmerone not only the structural but also the pharmacophoric analogue of DEET. Then the pharmacophore features of OBP1 (3OGN) interacting DEET and ar-turmerone were compared as represented in Fig. 2. For this the docked posed of DEET and ar-turmerone were superimposed and then their pharmacophore features were extracted in superimposed fashion. On doing so it was observed that, when interacting with OBP1, the aromatic pharmacophore feature (orange ring) of DEET and turmerone were identically overlapping, the feature of proton acceptor represented by inward arrow (in pink) and their bubbles (in grey) also overlapped, and lastly the bubbles (in grey) of hydrophobic pharmacophore (in green dots) also overlapped (Fig. 2). Thus, this shows that both the compounds, reference-DEET and lead-ar-turmerone interacts with OBP1 in identical fashion with identical spatial orientations along with their pharmacophore features occupying identical locus in the OBP1 protein cavity. Such in depth molecular interaction assessment proves ar-turmerone as a pharmacophoric analogue of DEET. Similar findings were obtained for OBP1 of *A. aegypti* (3K1E) in docking and in pharmacophore feature mapping (Fig. 3), showing for this protein too Trp114 plays significant role in the interaction with both, DEET and ar-Turmerone, the pattern of both these molecules occupying the binding cleft in the protein is identical and lastly, shows identical superimposition of the pharmacophoric features, thus consolidating the findings with two identical proteins. Further, comparison of chemical structure and features of DEET and ar-turmerone is represented in Table 2. Both the compounds have one proton acceptors and zero protein donor. The molecular weight and overall surface area of both the compounds are identical. Into their structures both have identical methyl containing terminal aromatic ring, a ketone functional group, and a branch at the other terminus making both these compounds a structural analogue of each other.

**Table 2:** Structural and chemical comparison of DEET and ar-turmerone.

Compound	DEET	ar-turmerone
Molecular structure		
Descriptor	Value	Value
Molecular weight	191.274	216.324
LogP	2.47702	4.02392
#Rotatable bonds	3	4
#Acceptors	1	1
#Donors	0	0
Surface area	85.542	98.188

### MD simulations and MM-GBSA assessment

MD simulations are serves as the backbone to validate the findings of docking assessment, where the simulations performed provides the surety check on the findings devised by molecular docking. The MD simulations will robustly determine the ligand’s interaction length, interaction types with the protein. Where docking only shows the type of interaction that may be exhibited by ligand with protein, MD simulations can compute the strength and duration of the inter-molecular interaction. Here the MD simulations of two complexes (i) DEET-OBP1 and (ii) ar-turmerone-OBP1 were performed for 50 ns and their outcomes were compared to assess the identity in the interaction pattern of both the ligands with OBP1.

**Figure 4:**
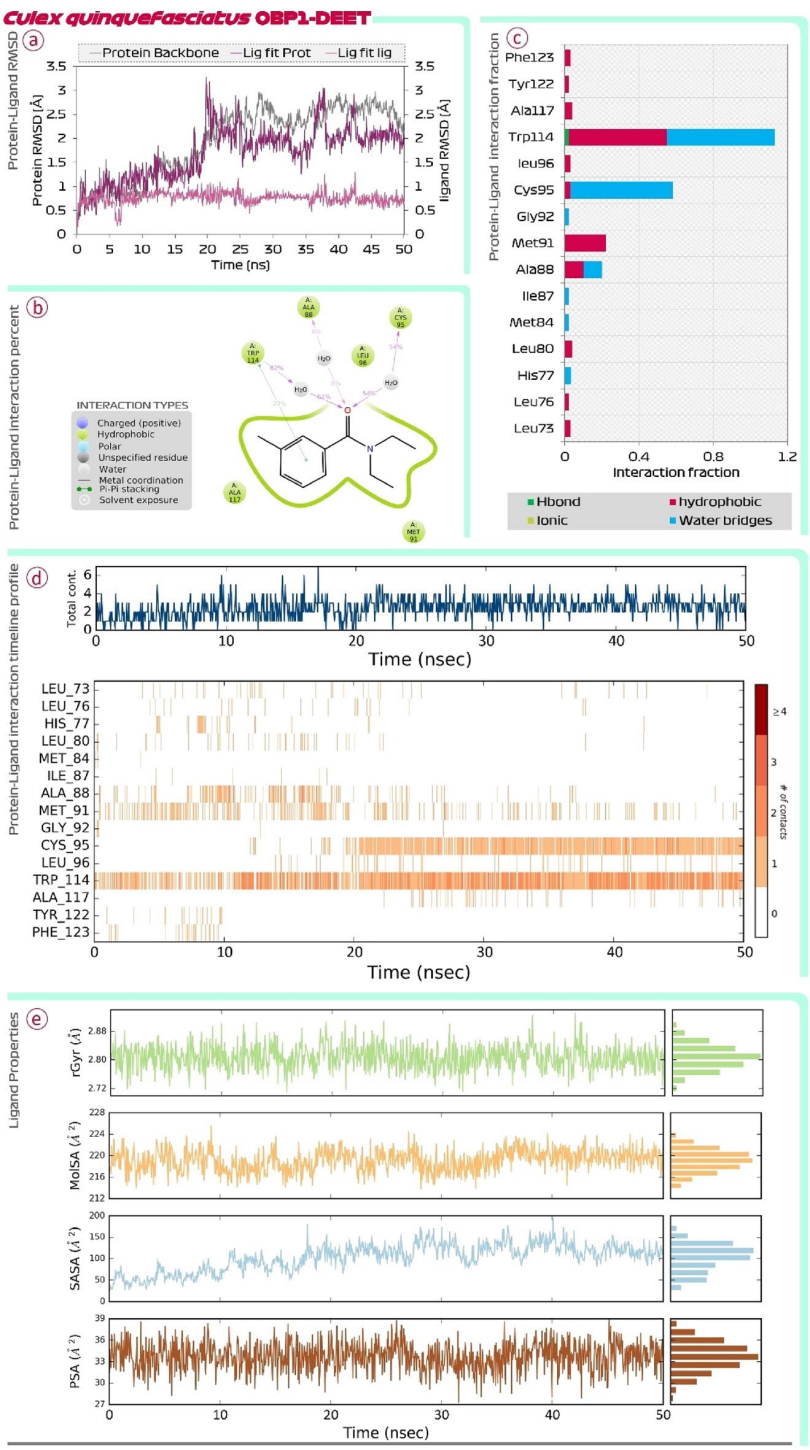
MD simulation assessment of DEET-OBP1 (3OGN) complex of *C. quinquefasciatus*, (**a**) RMSD profile, (**b**) percent interaction, (**c**) interaction fraction profile, (**d**) interaction timeline profile and, (**e**) Ligand properties during simulation run.

**Figure 5:**
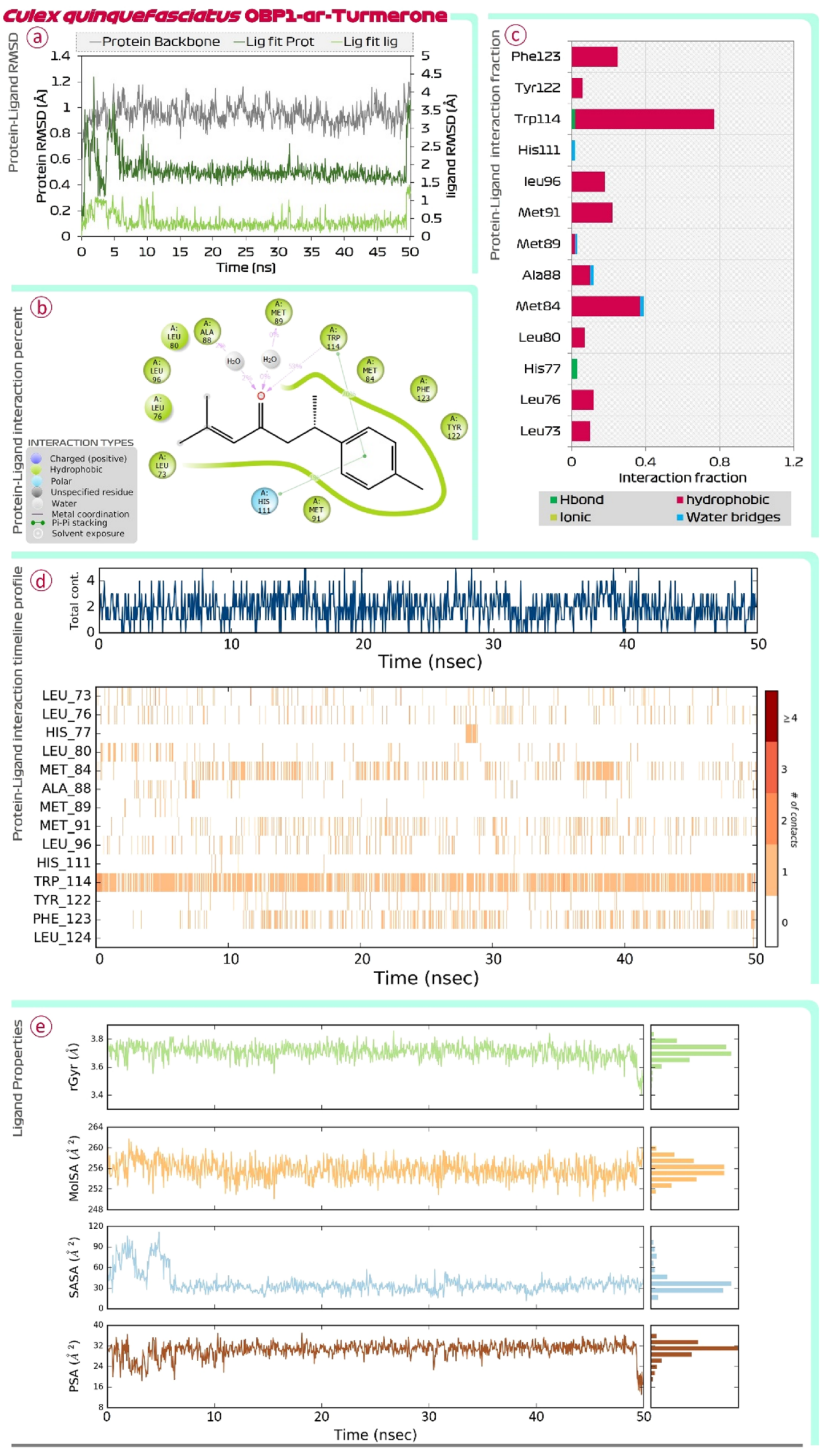
MD simulation assessment of ar-Turmerone-OBP1 (3OGN) complex of *C. quinquefasciatus*, (**a**) RMSD profile, (**b**) percent interaction, (**c**) interaction fraction profile, (**d**) interaction timeline profile and, (**e**) Ligand properties during simulation run.

**Figure 6:**
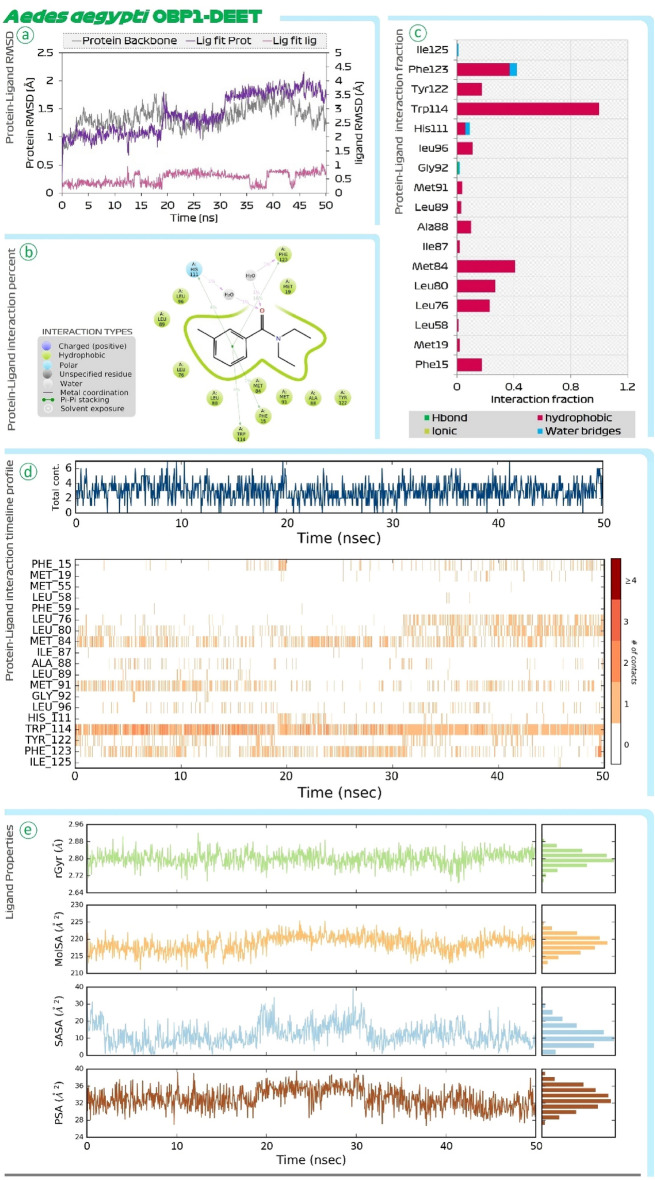
MD simulation assessment of DEET-OBP1 (3K1E) complex of *A. aegypti,* (**a**) RMSD profile, (**b**) percent interaction, (**c**) interaction fraction profile, (**d**) interaction timeline profile and, (**e**) Ligand properties during simulation run.

**Figure 7:**
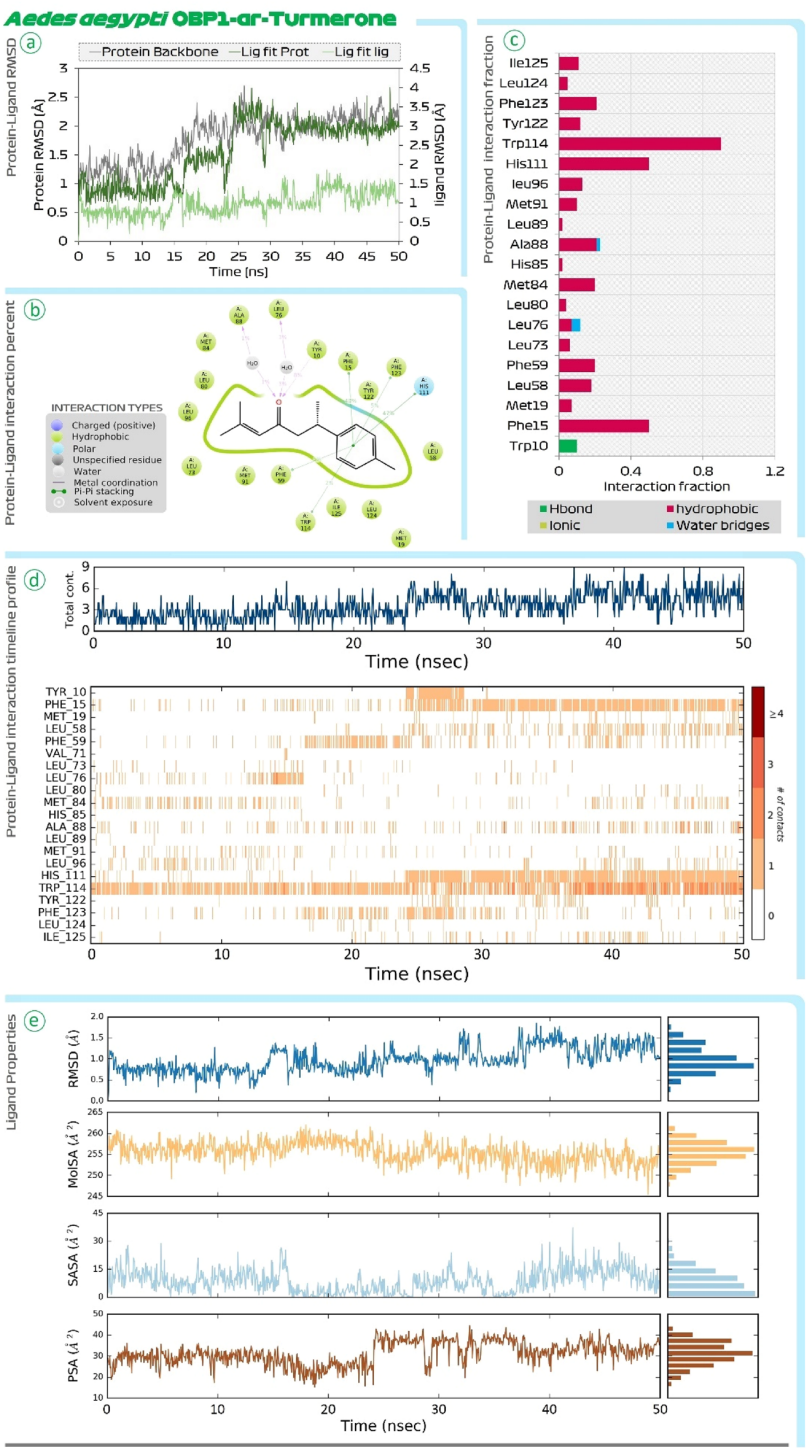
MD simulation assessment of ar-Turmerone-OBP1 (3K1E) complex of *A. aegypti,* (**a**) RMSD profile, (**b**) percent interaction, (**c**) interaction fraction profile, (**d**) interaction timeline profile and, (**e**) Ligand properties during simulation run.

Figure 4 shows the findings of MD simulations of DEET-OBP1 (3OGN) complex. Root Mean Square Deviations (RMSD) is the first assessment performed. In the RMSD plot there are two main assessments (i) Protein RMSD (ii) Ligand RMSD with respect to protein is to be compared. In the RMSD plot, the protein RMSD on the left Y-axis, variations in the range of 1 Å to 4 Å are totally reasonable; however, based on the different protein sizes, the range may expand. However, much larger changes indicate a significant structural shift in protein during MD recordings. In this case, for OBP1, the RMSD value never exceeds 3 Å, indicating that protein stability is satisfactory. The RMSD of the ligand, which is a measure of how stable the ligand (DEET) is bound at the catalytic site of OBP1, is shown on the right Y-axis. The value of 'Lig fit Prot', as this value doesn’t exceed considerably than the Protein RMSD, is acceptable. Similar findings of RMSD were also seen for the ar-turmerone-OBP1 complex represented in Fig. 5. Next, the interaction of DEET with OBP1 in detail be understood by protein ligand interaction summary image as shown in Fig. 4 and for ar-turmerone with OBP1 in Fig. 5, which shows interaction prevailing between ligand and protein during the MD simulation run, here it is observed that DEET and ar-turmerone both recruits Trp114 efficiently with their aromatic rings. Further it is observed that ketone group of DEET interacts with Ala88 and Cys95, while the same functional group of ar-turmerone interacts with Ala88 and Met89. Similar findings were consolidated when the MD simulations for both the ligands were repeated with OBP1 (3K1E) of *A. aegypti* where the MD simulation of OBP1-DEET is shown in Fig. 6 and OBP1-ar-turmerone is shown in Fig. 7.

Further, Protein–Ligand interaction fraction plot for both the complexes (i) DEET-OBP1 (Fig. 4) and (ii) ar-turmerone-OBP1 (Fig. 5) for 3OGN; while for 3K1E, DEET-OBP1 (Fig. 6) and (ii) ar-turmerone-OBP1 (Fig. 7) show, that both the interact with identical amino acids by making efficient interactions represented by interaction fraction values. For the plot, interaction fraction represents the value converted from percent interaction, viz. the interaction fraction of 0.4 suggests that 40% of the time of total MD simulation run time (50 ns), the interaction is formed. This value can be 1.0 which in 100% only for the covalent bond, as other interactions (H-bonds, hydrophobic interaction, ionic and water bridges) are dynamic in nature which has a very short life span of around 1 ps, so such bonds keep forming and breaking throughout the MD simulation run and its aggregate value is represented in the chart as interaction fraction. Further, total contact chats for both the complexes show that on an average the number of interactions by each ligand with OBP1 fluctuates making a lower average of two contacts and higher average of four contacts at any given instance during 50 ns MD simulation run. Lastly, protein–ligand interaction timeline chart for both the complexes represents instances when these dynamic bonds are formed and broken with respect to time. In the charts it is evident that DEET forms stable contacts with Trp114 throughout the simulation run (Figs. 4 and 6) and similar stable uniform interactions with same amino acid is also shown by ar-turmerone (Figs. 5 and 7). Such MD simulation study can well establish the interaction of ligands with protein receptors with great degree of fidelity and scientific fraternity with increasing computational power make use of this method to validate the ligand–protein interaction hypothesis. The behaviour of ligands, DEET (Figs. 4 and 6) and ar-turmerone (Figs. 5 and 7) during their interaction is represented by ‘ligand property’ charts. The ‘extendedness’ of a ligand and is equal to its essential snapshot of idleness is represented by radius of Gyration (rGyr) the 1.4 Å test sweep sub-atomic surface figuring is represented by Molecular Surface Area (MolSA). This worth is proportionate to a Van der Waals surface zone. Solvent Accessible Surface Area (SASA) is the surface zone of a molecule open for access to water molecules and finally, Polar Surface Area (PSA) is the dissolvable available surface territory in a particle contributed uniquely by oxygen and nitrogen atoms.

**Figure 8:**
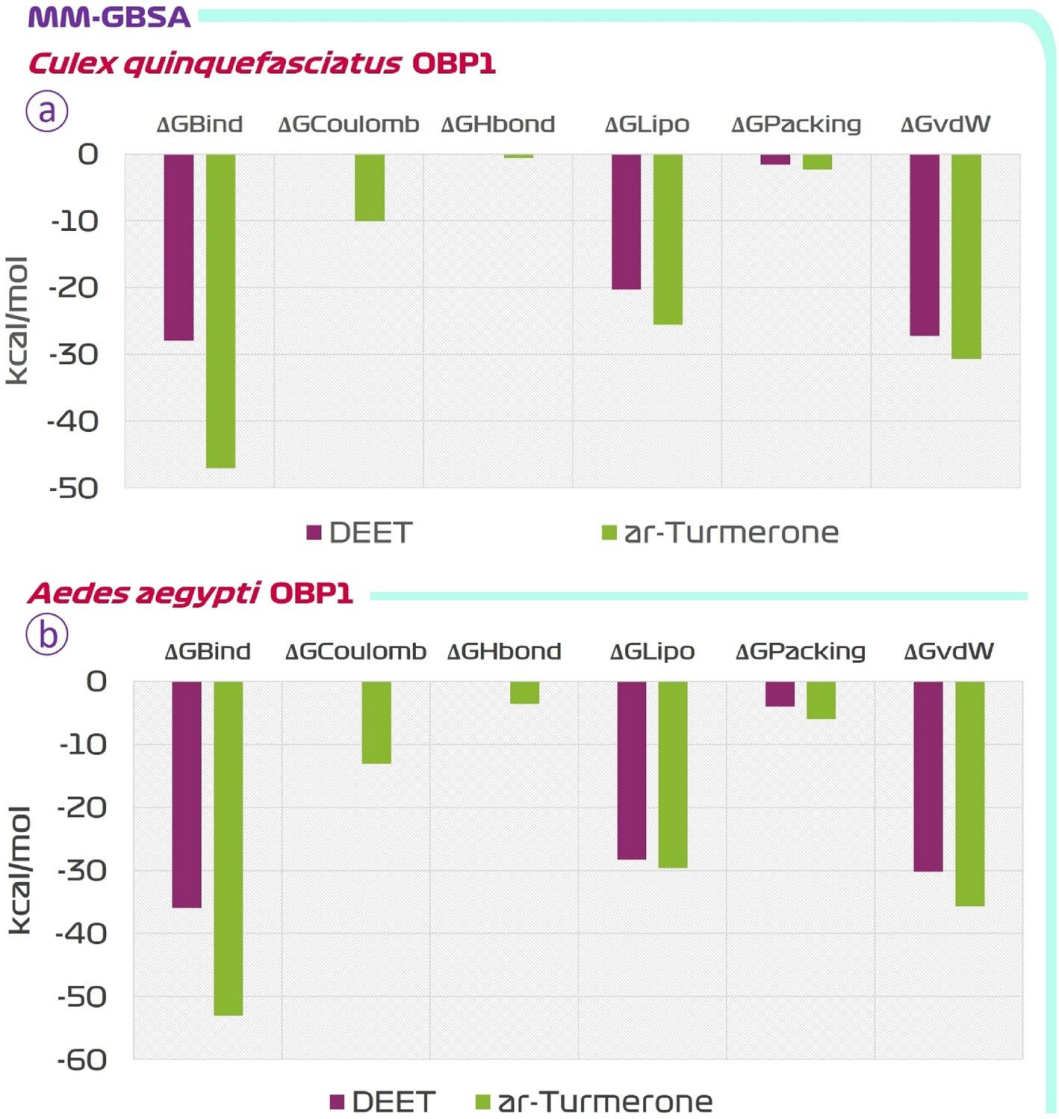
MM-GBSA assessment of DEET and ar-turmerone for their interaction propensity with OBP1 of (**a**) *C. quinquefasciatus,* and (**b**) *A. aegypti*, *ΔGBind* binding energy, *ΔGCoulomb* Coulomb energy, *ΔGHbond* hydrogen-bonding correction, *ΔGLipo* lipophilic energy, *ΔGPacking* Pi-Pi packing correction, *ΔGvdW* Van der Waals energy.

The MD trajectories obtained from simulations were used for assessing End-Point Binding energy change calculation by MM-GBSA post simulation assessment. This assessment provides more dependable and reliable values of ionic, hydrophilic, and hydrophobic attraction of the protein–ligand intricate. The ΔGbind energy is the Gibb’s free energy change conveyed from MM-GBSA assessment is the residual value when entropy value is subtracted from enthalpy, the value in negative range shows the interaction between ligand and protein is spontaneous. In short, a grater negative value indicates spontaneous binding and therefore ΔGBind (reported in kcal/mol) of MM-GBSA is used to estimate relative binding affinity of ligands. The ΔGbind profiles of DEET and ar-turmerone is represented in Fig. 8. For OBP1 (3OGN) the ΔGbind for DEET and ar-turmerone was found to be − 27 kcal/mol and − 44 kcal/mol respectively, similarly for OBP1 (3K1E) ΔGbind for DEET and ar-turmerone was found to be − 36 kcal/mol and − 53 kcal/mol respectively, thus suggesting that ar-turmerone has better spontaneity in interacting with OBP1 than DEET.

### ADMET assessment

The detailed predicted ADMET properties of DEET and ar-turmerone is represented in Table 3. The absorption properties for both these compounds are most important. Both these compounds have identical P-glycoprotein substrate, P-glycoprotein I inhibitor, and P-glycoprotein II inhibitor properties, where none of the compounds show reactivity or interaction to these proteins. The log Kp value for skin permeability assessment show that if this value is greater than 2.5 than it has low skin permeability, the log Kp for DEET is − 2.67 and for ar-turmerone is − 1.557, suggesting ar-turmerone has much lower skin permeability than that shown by DEET, making it much safter to use on skin. Other metabolism and distribution properties of both the compounds are identical. As both the compounds are not intended to be ingested or to be given as drug but are only intended to be use as mosquito repellent their discussion here won’t hold virtue to this manuscript, but the detailed value of each property is represented in Table 3.

**Table 3:** The ADMET properties of compounds under study.

Property	Model name	Compounds		Unit
		DEET	ar-turmerone	
Absorption	Water solubility	− 2.648	− 4.454	Numeric (log mol/L)
Absorption	Caco2 permeability	1.192	1.458	Numeric (log Papp in 10–6 cm/s)
Absorption	Intestinal absorption (human)	93.683	94.489	Numeric (% Absorbed)
Absorption	Skin Permeability	− 2.671	− 1.557	Numeric (log Kp)
Absorption	P-glycoprotein substrate	No	No	Categorical (Yes/No)
Absorption	P-glycoprotein I inhibitor	No	No	Categorical (Yes/No)
Absorption	P-glycoprotein II inhibitor	No	No	Categorical (Yes/No)
Distribution	VDss (human)	0.163	0.621	Numeric (log L/kg)
Distribution	Fraction unbound (human)	0.316	0.102	Numeric (Fu)
Distribution	BBB permeability	0.358	0.512	Numeric (log BB)
Distribution	CNS permeability	− 1.928	− 1.771	Numeric (log PS)
Metabolism	CYP2D6 substrate	No	No	Categorical (Yes/No)
Metabolism	CYP3A4 substrate	No	No	Categorical (Yes/No)
Metabolism	CYP1A2 inhibitor	Yes	Yes	Categorical (Yes/No)
Metabolism	CYP2C19 inhibitor	No	No	Categorical (Yes/No)
Metabolism	CYP2C9 inhibitor	No	No	Categorical (Yes/No)
Metabolism	CYP2D6 inhibitor	No	No	Categorical (Yes/No)
Metabolism	CYP3A4 inhibitor	No	No	Categorical (Yes/No)
Excretion	Total clearance	0.586	0.295	Numeric (log ml/min/kg)
Excretion	Renal OCT2 substrate	No	No	Categorical (Yes/No)
Toxicity	AMES toxicity	No	No	Categorical (Yes/No)
Toxicity	Max. tolerated dose (human)	0.818	0.846	Numeric (log mg/kg/day)
Toxicity	hERG I inhibitor	No	No	Categorical (Yes/No)
Toxicity	hERG II inhibitor	No	No	Categorical (Yes/No)
Toxicity	Oral rat acute toxicity (LD50)	2.311	1.843	Numeric (mol/kg)
Toxicity	Oral rat chronic toxicity (LOAEL)	1.46	1.11	Numeric (log mg/kg_bw/day)
Toxicity	Hepatotoxicity	No	No	Categorical (Yes/No)
Toxicity	Skin Sensitisation	Yes	Yes	Categorical (Yes/No)
Toxicity	*T. Pyriformis* toxicity	0.593	1.945	Numeric (log ug/L)
Toxicity	Minnow toxicity	1.194	0.005	Numeric (log mM)

## Discussion

Vector borne diseases (VBD) account for infecting diseases to over 80% of the world’s population residing in tropics and subtropics. Of all the known vectors, mosquitoes account for spreading deadly diseases such as dengue, chikungunya, Japanese encephalitis, malaria, yellow fever, filariasis, and zika virus infection which account for great degree of global mortality^31^. The transmission vector responsible for majority of the VBD’s are mosquitoes like *Aedes, Anopheles* and *Culex* belonging to the family of *Culicidae* of Order Diptera, known for spreading the infections for entirety of their life span^32^. Recent reports suggest that the transmission dynamics is about to get worse with the rise in the phenomena of global warming. Further, the climatic changes is predicted to increase the re-emergence of VBD’s by boosting the rate of development of the infectious pathogen within the vector itself, thus putting the mankind at a greater risk^33^. The easiest strategy for controlling the mosquitoes is by making use of mosquito repellents. Commonly used mosquito repellents are synthetic chemicals of noxious nature possessing tendency to cause allergy and toxicity upon their use by humans. Some widely used synthetic mosquito repellents include Benzaldehyde, Butopyronoxyl, Dimethyl carbate, DEET, Permethrin, Ethyl butrylacetylaminopropionate, etc. Of all DEET is one of the oldest and still widely used in multiple mosquito repellent formulations. DEET is known to interact with OBP1 and block signal transduction disabling them transporting odorants (like 1-octen-3-ol, a volatile substance that is contained in human sweat and breath) to olfactory receptors^10^. The pattern of interaction of DEET with OBP1 of mosquitoes is well studied and based on this information, researchers are pursuing to develop the analogues that can interact with OBP1 in an identical way^34^. The detail interaction profile of DEET with OBP1 of is represented by Tsitsanou et al. in the year 2012^6^ followed by Murphy et al. in the year 2013^7^. It was observed that DEET interacts with Trp114 of OBP1 which serves as important interaction for implying its action as mosquito repellent. Under current study, our XP docking results also showed DEET to interact with Trp114 of OBP1. Several bioinspired mosquito repellent namely (E)-3-(4-Methoxycarbonylphenyl)prop-2-enoate (KO9) is recently reported to act like DEET and shared structural similarity with Cumic acid is developed^34^.

Synthetic mosquito repellents being noxious in nature and overuse of these chemicals have caused mosquitoes to become resistance towards their repellent activity, therefore researchers are making leaps in finding natural alternative to synthetic repellents. With advancement in bioinformatic tools and compound libraries, compounds such as carvacryl acetate, thymyl isovalerate, thymol acetate, 4-(4-methylphenyl)-pentanal, p-anisyl hexanoate and p-cymen-8-yl from the libraries of natural volatile compounds from essential oils are screened having potency to interact with OBP1^35^. But till date none of these compounds have been identified as analogue of DEET. On the other hand, essential oil from *C. longa* plant is well perceived to have mosquito repellent potentials with multiple promising publications and has been used by ayurveda to be a natural mosquito repellent. But till date there are no evidence for its phytochemicals interacting with OBP1 to induce its action as repellents. Therefore, to fill this knowledge gap we under current study constructed library of all the known phytochemicals from *C. longa* and compared their binding activity with OBP1 keeping DEET as reference. To the best of our knowledge this is the first report examining molecular interactions of volatile compounds with OBP1 of mosquito. From our study we found a compound ar-turmerone to bind efficiently with OBP1, but it recruited all the essential amino acids including Trp114 that are essential to cause inhibition of OBP1 as previously reported^6^. Current manuscript is the first in silico report proving its effective interaction with OBP1 but Ali et al. in the year 2015 have proved that ar-turmerone can serve as an efficient mosquito repellent and has potency at par with DEET^29^. In their study, they reported ar-turmerone to have the biting deterrent activity higher than DEET at when applied on skin at a concentration of 25 nmol/cm^2^ whereas the activity of other curcuminoids were lower than DEET^29^. In their article there is a strong claim of ar-turmerone being even better than DEET. However, there study lacked molecular comparison study which we did in this study. With this observed information, it can be stated that ar-turmerone and DEET are functional analogues where both serve as mosquito repellents.

Next, we on knowing this we compared the docked poses of DEET and ar-turmerone where we found that spatial arrangement where both these compounds interact with OBP1 is identical. So, we decided to compare the pharmacophore features of both these compounds Typically, these features include are classified into hydrophobic centroids, aromatic rings, hydrogen bond acceptors or donors, cations, and anions^36,37^. Under current study DEET is found to have three hydrophobic centres, one aromatic feature and one hydrogen acceptor feature. In the domain of cheminformatics, pharmacophore analogues are compounds that that share essential pharmacophore features making them exhibit same biological activity^38^. When curcuminoids were screened to check the identity in the pharmacophore features with DEET, none but only ar-turmerone had all the identical features to that of DEET. Further, when the docked pose of DEET and ar-turmerone while interacting with OBP1 were extracted, the pharmacophores of both the compounds occupy identical locus in the active site of OBP1 and these features even superimposed identically. Moreover, both the compound shares structural similarity to a great degree. Lastly, the interaction of DEET and ar-turmerone with OBP1 was simulated at psychological conditions using MD simulations. Such MD simulation study can well establish the interaction of ligands with protein receptors with great degree of fidelity and scientific fraternity with increasing computational power make use of it to validate the hypothesis^39–46^. Findings of MD simulation consolidated the predictions of docking that ar-turmerone interacts with OBP1 at par with DEET. Thus, with this it can be for the first time deduced that ar-turmerone is the structural, functional and pharmacophoric analogue of synthetic chemical repellent DEET.

## Materials and methods

### C. longa ligand library preparation and library energy minimization

All the possible phytochemicals that are known to be present in *C. longa* was identified from IMPPAT database by selecting plant *C. longa* in 'phytochemical composition” selection of ‘browse’ wizard which identified 15 phytochemicals namely ‘dihydrocurcumin, bisdemethoxycurcumin, ar-turmerone, tetrahydro bisdemethoxycurcumin, piperine, zingiberene, 2-methylisoborneol, butylated hydroxytoluene, borneol, demethoxycurcumin, sabinene, alpha-phellandrene, butylhydroxyanisole, cineol and curlone. Along with the names of these phytochemicals, their PubChem CID numbers were also provided, based on this CID all the phytochemicals were retrieved in the SDF format from PubChem database. The reference repellent DEET was also retrieved from PubChem (CID: 4284). All these molecules (phytochemicals and DEET) were then imported to Schrödinger Maestro for ligand preparation which is an inevitable step prior to molecular docking assessment. Ligand preparation helps in generating the low energy structures and allow the option to expand each input’s structure according to its desired stereochemistry by generating variations on ionisation state tautomer’s ad ring confirmations. LigPrep wizard in Schrödinger Maestro was used to generate ionization states for each ligand structure with Epik^47,48^ at a physiological pH of 7.2 ± 0.2 unit. Rest other options were kept as default and the ligands were minimized under OPLS2005 force field. The output files so obtained on performing ligand minimization was used for performing Extra precision (XP) docking and for E-pharmacophore feature mapping in Schrödinger Maestro.

### OBP1 retrieval and its preparation

Crystal Structure of an Odorant-binding Protein (OBP1) from the southern house mosquito (*C. quinquefasciatus*) complexed with an oviposition pheromone ((1S)-1-[(2R)-6-oxotetrahydro-2H-pyran-2-yl]undecyl acetate) was retrieved from Protein Databank (PDB) having PDB ID: 3OGN. From its X-ray Diffraction analysis data, resolution was found to be1.30 Å, R-value free was identified to be 0.174, R-Value work was 0.132 and R-Value observed was 0.134. Same protein from another mosquito, *A. aegypti* was retrieved from PDB having ID: 3K1E. From its X-ray Diffraction analysis data, resolution was found to be1.85 Å, R-value free was identified to be 0.212, R-Value work was 0.151 and R-Value observed was 0.134. The size of both the proteins were identical having following dimensions, 35.915 Å × 107.31 Å × 38.529 Å. Both proteins were imported to Schrödinger Maestro and were prepared individually by using of Maestro’s ‘Protein preparation wizard’. Initially, protein was pre-processed by adding hydrogens, converting selenomethionines to methionine and heterogenous states were generated Epik for pH 7.0 followed to this as a next step in protein preparation, h-bond assignment was done using PROPKA for pH 7.0 for optimizing the protein. On achieving successful protein preparation, it was then optimized, the protein restrained minimization was done using OPLS-2005 (Optimized Kanhesia for Liquid Simulations) force field^49–51^. All these tasks were performed in Schrödinger Maestro ‘Protein Preparation Wizard’^52,53^. Once these steps were successfully incorporated, i.e., pre-processed, optimized and minimized OBP1 was now ready to be used for the next step of molecular docking.

### Molecular docking

Both the prepared OBP1s from the previous step was used for docking assessment. The first step for docking is to prepare the grid box at the exact same co-ordinates as that of native ligand found in both the proteins 3OGN and 3K1E. The size of the grid box 10 Å × 10 Å × 10 Å so prepared at the co-ordinates with the centre position defined as, 3.49° on X axis, 30.69° on Y axis and 9.51° on Z axis. ‘Receptor grid generation’ wizard of Schrödinger Maestro Glide was used to prepare grid for docking. The output file of (i) prepared minimized ligands (all *C. longa* phytochemicals and DEET) from previous step and (2) receptor grid file was imported in the ‘Ligand docking’ wizard of Glide module in Schrödinger Maestro. Under the settings, the precision of docking was set to ‘Extra Precision (XP)’, ligand sampling was set to ‘flexible’ and the Epik state penalties were added to docking score. The output was set to show only the best pose. The entire docking was performed using Schrödinger Maestro’s Glide module^52,53^. On performing XP docking, the DEET docked to OBP1 was taken as reference control for all the following assessments.

### E-pharmacophore feature mapping

As DEET is reference synthetic chemical repellent used under study, we intended to identify the analogous molecule from *C. longa* which as similar chemical structural features and to accomplish this agenda, E-pharmacophore feature mapping assessment was used. For this, all the ligands were imported to Schrödinger Maestro and DEET was selected to generate its pharmacophore features making use of ‘Develop Pharmacophore model’ wizard in Phase module of Schrödinger Maestro. In the settings, all possible features were included in developing the hypothesis. Followed to this, the pharmacophore hypothesis of DEET so generated was used to screen all the phytochemicals of *C. longa* to identify its pharmacophoric analogue and to accomplish this, ‘Ligand Based Screening’ wizard of Phase module was used. Once the ligands were screened, the pharmacophore feature superimposition assessment was performed, here the pharmacophore features of DEET while interacting with OBP1 complex was extracted and similar was also done for the pharmacophoric analogue. These features of each ligand (DEET and identified pharmacophoric analogue) interacting with OBP1 were superimposed to determine the identity in latency of molecular ability to interact with OBP1.

### Molecular dynamic (MD) simulation

The complexes (i) DEET-OBP1 and (ii) selected phytochemical-OBP1 were subjected to MD simulation 50 ns each using Desmond package (Schrödinger Release 2018-4)^54^. Prior to subjecting complexes to MD simulations, their energy minimization was done by OPLS-2005 force field, after which the system was build using TIP3P solvent model. Simulation box of orthorhombic shape fitting the protein ligand complex was prepared with 15 Å buffer space around ligand–protein complex. Simulation box neutralisation was then performed to simulate the background salt and physiological conditions by placing Na + ions and salt concentration of 0.15 M Na + and Cl− counter ions using OPLS-2005 force fields. On successful building up of the system, MD simulation was performed with NPT (constant Number of particles, Pressure, and Temperature) ensemble with 300 K temperature and 1.013 bar atomic pressure and default surface tension using Smooth Particle Mesh Ewald (PME) method is used to calculate long range electrostatic interaction potential energies. The 50 ns MD simulation was executed and 1000 frames of trajectories were recorded. On completion of simulation, each trajectory was analysed in Simulation Interaction Diagram wizard which computes trajectories for Root Mean Square Deviation (RMSD) and Root Means Square Fluctuation (RMSF). Protein–ligand contact profiles for crucial interacting amino acid residues and timeline of these specific interactions are also computed with respect to 50 ns simulation. The validation of docking poses, and interactions predicted by both the ligands with OBP1 during docking was validated using this procedure of MD simulations.

### MM-GBSA calculation

The Gibb’s free energy change, ΔG (kcal/mol) was computed using Molecular Mechanics Generalized Born Surface Area (MM-GBSA) for the interaction of DEET and its identified analogue with OBP1^55–58^. The output file of MD simulation so obtained was used for MM-GBSA calculations. The file was imported to the Prime wizard of Schrödinger Maestro's where it was optimized^59^. The free energy change on binding ΔG, computed by OPLS-2005 force field. The ΔG binding energy transition was calculated using the following equation:

1 $$\Delta {\text{GBind}} = \Delta {\text{EMM}} + \Delta {\text{GSolv}} + \Delta {\text{GSA}}$$

Here, ΔGBind stands for the binding of receptor and ligand molecules in solution as the molar Gibbs energy. ΔEMM is the variance between the minimized energy of the protein–ligand complexes, while ΔGSolv is the sum of the solvation energies for the protein and ligand and the variation between the GBSA solvation energy of the same. ΔGSA is the difference in the surface area energies for the complexes. After assessing the docking score and ΔGBind score of MM-GBSA the latent ability of DEET and its analogue to interact spontaneously with OBP1 was compared.

### Pharmacokinetics assessment using ADMET computational assessment

Toxicity of DEET is sought to be high and therefore an alternate molecule that is proposed to a DEET-analogous should have lower toxicity on human body and therefore toxicity of the reference and test compound should be evaluated. For this, ADMET (Absorption, Distribution, Metabolism, and Excretion) assessment can be performed computationally which can predict the pharmacokinetics of the compounds under study. Pharmacokinetic properties for DEET and its identified natural analogue is predicted using pkCSM- pharmacokinetics server^60^. For such assessment, SMILES (Simplified Molecule Input Line Entry Specification) from PubChem were retrieved for compounds under study and were fed on to the pkCSM-pharmacokinetics server. As none of the compound under study is directly ingested but are applied on skin, so there is high chance that it can penetrate skin and show skin toxicity, and this is well reported for DEET, therefore ADMET assessment holds virtue even though such mosquitos’ repellents are not directly ingested.

Both pharmacokinetic and toxicity properties were predicted using SMILES (Simplified Molecule Input Line Entry Specification) retrieved from PubChem for the lead compounds. This server compute in vivo Absorption parameters like; Water solubility (SK atomic types, mg/L), in vivo Caco2 cell permeability (Human colorectal carcinoma), Human intestinal absorption (HIA, %), in vivo P-glycoprotein substrate, inhibitor I & II, and assessment of in vivo skin permeability (log Kp, cm/hour). Distribution property included tests like, Volume of Distribution of drug in the human system (VDss (human)), Plasma Protein fraction unbound feasibility, Blood–Brain Barrier (BBB) permeability and Central Nervous System (CNS) penetration. Metabolic parameters were determined using *in-vivo* Cytochrome P450 2C19, Cytochrome P450 2C9, Cytochrome P450 2D6, Cytochrome P450 3A4 inhibition, along with in vivo Cytochrome P450 2D6 and 3A4 substrate inhibition. Total Renal clearance and Renal OCT2 Substrate is predicted to identify Excretion efficacy. Toxicity assessments are also predicted including Acute algae toxicity, Ames test of mutagenicity, two years carcinogenicity bioassay in mouse, two years carcinogenicity bioassay in rat, *in-vivo* Ames test result in TA100 strain (Metabolic activation by rat liver homogenate).

## Data Availability

All the relevant data is contained within the manuscript. Additional raw data will be available upon request.
